# Particulate matter (PM_10_) induces in vitro activation of human neutrophils, and lung histopathological alterations in a mouse model

**DOI:** 10.1038/s41598-022-11553-6

**Published:** 2022-05-09

**Authors:** Andrés Valderrama, Paul Ortiz-Hernández, Juan Manuel Agraz-Cibrián, Jorge H. Tabares-Guevara, Diana M. Gómez, José Francisco Zambrano-Zaragoza, Natalia A. Taborda, Juan C. Hernandez

**Affiliations:** 1 Infettare, Facultad de Medicina, Universidad Cooperativa de Colombia, Medellín, Colombia; 2 Unidad Académica de Ciencias Químico Biológicas y Farmacéuticas, Universidad Autónoma de Nayarit, Tepic, Nayarit México; 3 Grupo de Investigaciones Biomédicas Uniremington, Programa de Medicina, Facultad de Ciencias de La Salud, Corporación Universitaria Remington, Medellín, Colombia

**Keywords:** Innate immune cells, Innate immunity, Environmental impact, Biological techniques

## Abstract

The epidemiological association between exposure to particulate matter (PM_10_) and various respiratory and cardiovascular problems is well known, but the mechanisms driving these effects remain unclear. Neutrophils play an essential role in immune defense against foreign agents and also participate in the development of inflammatory responses. However, the role of these cells in the PM_10_ induced inflammatory response is not yet fully established. Thus, this study aims to evaluate the effect of PM_10_ on the neutrophil-mediated inflammatory response. For this, neutrophils from healthy adult human donors were in vitro exposed to different concentrations of PM_10_. The cell viability and cytotoxic activity were evaluated by MTT. LDH, propidium iodide and reactive oxygen species (ROS) were quantified by flow cytometry. Interleukin 8 (IL-8) expression, peptidyl arginine deiminase 4 (PAD_4_), myeloperoxidase (MPO), and neutrophil elastase (NE) expression were measured by RT-PCR. IL-8 was also quantified by ELISA. Fluorescence microscopy was used to evaluate neutrophil extracellular traps (NETs) release. The in vivo inflammatory responses were assessed in BALB/c mice exposed to PM_10_ by histopathology and RT-PCR. The analysis shows that PM_10_ exposure induced a cytotoxic effect on neutrophils, evidenced by necrosis and LDH release at high PM_10_ concentrations. ROS production, IL-8, MPO, NE expression, and NETs release were increased at all PM_10_ concentrations assessed. Neutrophil infiltration in bronchoalveolar lavage fluid (BALF), histopathological changes with inflammatory cell infiltration, and CXCL1 expression were observed in PM_10_-treated mice. The results suggest that lung inflammation in response to PM_10_ could be mediated by neutrophils activation. In this case, these cells migrate to the lungs and release pro-inflamatory mediators, including ROS, IL-8, and NETs. Thus, contributing to the exacerbation of respiratory pathologies, such as allergies, infectious and obstructive diseases.

## Introduction

Air pollution represents a major threat to human health^1^. According to the World Health Organization (WHO), exposure to air pollutants is the cause of 4.2 million premature deaths per year^2^. This mortality is related to adverse respiratory and cardiovascular health effects caused by exposure to particulate matter (PM)^3,4^.

Particulate pollutants are originated from the natural environment (volcanoes, forest fires and dust storms) and human activities (transportation, industry, power plants, combustion, and agriculture)^5^. According to their aerodynamic diameter, they are classified into particles including PM_10_ (less than 10 µm); PM _2.5_ (less than 2.5 µm), and ultrafine particles (UFP, less than 0.1 µm)^6,7^. PM can penetrate the human respiratory tract and even reach circulation due to its small size, large surface area, penetration capacity, deposition, bioavailability, and long residence time in the air^8^. Exposure to PM can induce lung damage by oxidative stress and airway inflammation^9^, loss of immune functions against microorganisms^10,11^, thrombosis, coagulation, and vascular dysfunction that can lead to the development of different diseases^12–14^. Epidemiological and toxicological studies related to PM have shown positive correlations with the development of a wide range of diseases, including skin diseases^15^, asthma^16^, stroke, and ischemic heart disease^17^, chronic obstructive pulmonary disease (COPD)^18^ and lung cancer^19^.

Although the pathogenesis of diseases produced by PM exposure is not completely clear, several studies have demonstrated mechanisms potentially associated with innate and adaptive immune alterations. These processes include cytotoxicity, production of cytokines and pro-inflammatory molecules, mutagenicity, oxidative DNA damage, and genotoxicity^20^. Inflammatory responses activated by PM exposure may underlie asthma, chronic obstructive pulmonary disease, and lung cancer^10,21^.

Neutrophils constitute the most considerable fraction of leukocytes in the human body, with an essential role in lung immune response. These cells rapidly migrate to the lung, destroy the foreign agent and initiate an inflammatory response predominantly mediated by phagocytosis, ROS production, degranulation, and NETosis^22^. Neutrophil extracellular traps (NETs) are produced by the extracellular release of neutrophil nuclear material coated with antimicrobial peptides and enzymes, mainly activated by ROS production. NETosis activation is associated with exacerbated inflammatory response, tissue damage, and potentially, autoimmunity diseases^23–25^. Consequently, PM inhalation and deposition could induce ROS and oxidative stress molecules that induce neutrophil activation, NETs overproduction, and exacerbated inflammatory response.

Different in vivo studies have evidenced that PM exposure promotes neutrophil migration and lungs infiltration. Asthmatic BALB/c mice exposed to different concentrations of PM_2.5_ exhibited a significant increase in the frequency of eosinophils and neutrophils and high production of TNF-α in bronchoalveolar lavage fluid (BALF). Aberrant accumulation and altered functions of neutrophils in the lung are related to increased inflammation and tissue damage in people exposed to PM^26^. In this regard, it has been shown that neutrophils activated by PM exposure are accumulated in the pulmonary vasculature and induce an elevated release of inflammatory mediators such as myeloperoxidase (MPO)^27^ and leukotriene B4 (LTB_4_)^28^. Similarly, an in vitro study showed that neutrophil exposure to PM increased the release of (LTB_4_), leukotriene C4 (LTC_4_), and IL-8^29^. These mediators may be responsible for oxidative and proteolytic tissue damage, leading to immune dysregulation and lung diseases.

Although different studies provide convincing evidence regarding PM exposure-related immune alterations, relatively few studies are available in the context of neutrophil-mediated inflammatory responses. Therefore, this study aimed to determine the in vivo and in vitro effect of PM_10_ on the neutrophil-mediated inflammatory response.

## Materials and methods

### Ethics statement

The study was performed according to the principles of the declaration of Helsinki and approved by the Ethical Committee of the Universidad Cooperativa de Colombia (certificate number 003/2018). The individuals enrolled provided signed informed consent forms.

Experiments in mice were approved by the corresponding Institutional Animal Care and Use Committees (CICUA), Universidad de Antioquia (certificate number 117), and performed following international guidelines and regulations.

The manuscript follows the recommendations in the ARRIVE guidelines.

### Neutrophil isolation and culture

Neutrophilic polymorphonuclears (PMNs) were isolated from freshly drawn peripheral venous blood from healthy humans. Cells were separated by centrifugation at 3000 r.p.m for 40 min at room temperature in a double density gradient Ficoll/Histopaque 1119 and 1077 (Histopaque 1119 and 1077, Sigma—UK). The layer containing PMNs was then collected, washed twice with phosphate-buffered saline (PBS), and subsequently resuspended in RPMI medium (RPMI 1640, Gibco-USA) supplemented with 10% fetal bovine serum (FBS) (Sigma-Aldrich) and 1% penicillin–streptomycin (GIBCO Invitrogen, Breda, The Netherlands). Cell viability after isolation and before the start of each experiment was > 95%, as measured by the trypan exclusion test.

### PM_10_ collection

PM_10_ was collected in Valle de Aburrá, Colombia, between January-June of 2018, due to the most intense air pollution episode of the year, and using the local environmental authority of Valle de Aburrá (Sistema de Alerta Temprana—SIATA). The PM_10_ sample was obtained from 100 quartz filters at ten BAM-1020 monitoring stations (TISCH Environmental, BM2000H). The PM_10_ filters were cut into small pieces; high purity sterile water was added, mixed by immersion, and sonicated for 2 h and 15 min at 37 Hz. The mixture was then filtered using six layers of sterile gauze. After distributing the collected solution into sterile vials and freeze-drying under a vacuum, the tube was weighed to determine the mass of the extracted particles and stored at 80 °C before biological testing. A vacuum lyophilizer (ALPHAI-2LDplus, Martin Christ, Osterode, Germany) was used to dry the PM_10_ to particulate powder.

### Cell culture and treatment

PMNs (2.5 × 10^5^ cells/well) were seeded in 96-well plates (Greiner-Bio-One, Solingen, Germany). Increasing concentrations (0.1, to 100 µg/mL) of PM_10_ were prepared in RPMI 1640 medium. Subsequently, the freshly prepared PM_10_ solutions were added to the cells at a maximum volume of 200 µL per well. After exposure at 37 °C and 5% CO_2_ for 5 h, the supernatant was removed and used for LDH analysis or stored at − 80 °C until further analysis (cytokine levels by ELISA). Cell pellets were used for total RNA extraction and MTT assay. As positive controls, we used cells stimulated with the following stimuli: MTT assays and LDH assay: 20% dimethyl sulfoxide (DMSO); RT-PCR and ELISA: 50 ng/mL lipopolysaccharide (LPS) (Invitrogen, San Diego, CA); flow cytometry: Zymosan; fluorescence microscopy: 50 nM Phorbol-12-myristate-13-acetate (PMA). In all assays, PM_10_ unexposed cells were used as a negative control. All reagents and materials used in the experiments were endotoxin-free.

### Cytotoxicity assays

PM cytotoxic effect was evaluated using the colorimetric 3-(4,5-dimethylthiazol-2-yl) diphenyltetrazolium bromide (MTT) reduction, Lactate Dehydrogenase (LDH) release, and propidium iodide (PI) staining assays.

For MTT assay, PMNs were exposed to different concentrations of PM_10_ (0.1, 1, 10, 50, and 100 µg/mL) and 20% DMSO for 5 h. The supernatant was removed from each well, and the cells were washed with PBS. Then, the cells were incubated in a fresh medium containing 0.5 mg/mL MTT for 3 h at 37 °C. The formed crystalline formazan was dissolved in 100 µL DMSO. Finally, absorbance was measured at 570 nm using a microplate reader (Multiskan™ FC Microplate Photometer, Thermo Scientific). The data were normalized to untreated control cells.

LDH release in the PMNs culture medium was measured using the LDH toxicity assay kit (Roche, Germany) in supernatants of three different culture conditions: untreated PMNs (low control), treated with DMSO (high control) and exposed to different concentrations of PM_10_. After 5 h of culture, supernatants were transferred to a 96-well plate in triplicate and incubated for 30 min at room temperature with the reaction mixture. The final absorbance was measured at 490 nm using a microplate reader (Multiskan™ FC Microplate Photometer, Thermo Scientific). Cytotoxicity was calculated as: % cytotoxicity = [(LDH activity with stimulus (PM_10_)-(LDH activity low control)/(LDH activity high control-LDH activity low control)] × 100%.

For Propidium iodide (PI) staining assays (for necrosis), PMNs were cultured at a density of 1 × 10^6^ on round coverslips and treated with different concentrations of PM_10_ (10, 50, and 100 µg/mL) for 30 min at 37 °C. The cells were washed, stained for 1 h, and observed with a fluorescent microscope.

### RNA extraction and quantitative real-time PCR

IL-8, PAD_4_, NE, and MPO mRNA levels in PMNs exposed to different concentrations of PM_10_ (0.1, 1, 10, 50, and 100 µg/mL) and LPS (50 ng/ml) were determined by real-time PCR. Total RNA was isolated using the One-Step RNeasy Mini Kit extraction kit, according to the manufacturer's recommendations (Qiagen, Hilden, Germany). RNA concentration was quantified using a Nanodrop one spectrophotometer (Thermo Scientific). RNA was reverse transcribed into cDNA in a 20 μL reaction volume using the iScript High Capacity cDNA Reverse Transcription Kit, following the manufacturer's recommended protocol (Bio-Rad Laboratories, Hercules, CA). The qPCR was performed using SYBR Green Mastermix (Thermo Scientific, Waltham, MA) on a QuantStudio 3 real-time PCR detection system (Applied Biosystems). The list of primer sequences used to detect mRNAs is presented in Table 1. Relative gene expression levels obtained from reverse transcription qRT-PCR were calculated using the ΔΔCt method and normalized to phosphoglycerate kinase (PGK) gene expression.

**Table 1:** Primers.

Gen	Primers 5'-3'	Annealing temperature
IL-8	Fw: 5′-ACTGAGAGTGATTGAGAGTGGAC-3′ Rv: 5′-AACCCTCTGCACCCAGTTTTC -3′	60 °C
PAD_4_	FW: 5'-GGGGTGGTCGTGGATATTGC-3 ′ Rv: 5'-CCCGGTGAGGTAGAGTAGAGC-3 ′	64 °C
NE	FW: 5′-GTGGCGAATGTAAACGTC-3 ′ Rv: 5′-CCGTTGAGCTGGAGAATC-3′	58 °C
MPO	Fw: 5′-TGCTTCCTGGCAGGGGA-3′ Rv: 5′-CCACCTAG GGTTCAGGCTCT-3′	62 °C
PGK	Fw: 5′-GTTGACCGAATCACCGACC -3′ Rv: 5′-CGACTCTCATAACGACCCGC -3′	60 °C

### Cytokine analysis

To investigate the effect of PM_10_ on IL-8 production, the concentration of this cytokine in cell culture supernatants was determined using a commercial ELISA assay (BioLegend, San Diego, CA) according to the manufacturer's instructions. All samples were performed in triplicate. Cytokine concentrations were calculated from a standard curve of the corresponding recombinant human cytokine.

### NETs induction

Round coverslips were treated with ethanol and poly-L-lysine (Sigma) then placed in a 12-well plate with PBS. 5 × 10^5^ PMNs were added per well with the following stimuli: (a) PMA as positive control; (b) 10 µg/ml of PM_10_, (c) 50 µg/ml of PM_10_, and (d) 100 µg/ml of PM_10_, PBS was used as a negative control. The cells were incubated without FBS for 3 h at 37 °C in the presence of 5% CO_2_, then fixed with 1 mL of 4% paraformaldehyde for 20 min at room temperature. Cell's DNA was stained with 4′,6-diamidino-2-phenylindole (DAPI) and incubated at 4 °C overnight.

Afterward, cells were permeabilized with Triton X-100 and blocked with 1% BSA for 30 min at 37 °C. Then 1 mL of anti-neutrophil elastase (1:500, Abcam) and anti-myeloperoxidase (1:500, Abcam) primary antibodies were added to each well, and the plate was incubated for 1 h at 37 °C. 1 mL of anti-rabbit IgG (1:1000, Abcam) Alexa 488 and anti-rabbit IgG (1:500, Abcam) Alexa 647 secondary antibodies were then applied, the plate was incubated for 1 h at 37 °C. DNA, Neutrophil elastase (NE), and Myeloperoxidase (MPO) were identified by immunofluorescence. Images of random fields were taken through the 20 × and 40 × objectives. Each experiment was repeated at least three times.

### Quantification of ROS production

Intracellular ROS production in PMNs exposed to PM_10_ was measured with the DHR-_123_ ROS dihydrorhodamine assay kit (Thermo Fisher Scientific, USA), according to the manufacturer's protocol. Briefly, PMNs (5 × 10^5^ cells) were cultured in flow cytometry tubes (BD Falcon polystyrene) and exposed to different concentrations of PM_10_ (0.1, 1, 10, and 50 µg/mL) and zymosan (10 µg/mL) for 30 min. An unstimulated control tube was included in each assay. Cells were then washed with PBS, and 100 μL PBS supplemented with DHR-123 at a concentration of 0.07 μg/mL was added and incubated at 37 °C for one hour. Cells were then washed and resuspended in 200 μL of PBS. The analysis was performed on a BD LSR Fortessa™, and the collected data were analyzed in FlowJo v10.4 software (FlowJo, LLC). The neutrophil population was classified by forward scatter (FSC), and side scatter (SSC), where at least 10,000 events were recorded. Doublets were excluded by forward scatter height (FSC-H) and forward scatter area (FSC-A). FL-1 channel mean fluorescence (FITC) was examined for an increase in Rho123 fluorescence representing ROS production in activated versus unstimulated cells (Fig. S1). ROS production was calculated by subtracting the percentage of ROS production of stimulated PMNs from the percentage of ROS of control PMNs.

### Mice

8-weeks-old male wild-type BALB/c mice weighing 18–20 g (Charles River, Portage, MI, USA) were bred and housed at 22 ± 1 °C under a 12 h light/dark cycle with food and water ad libitum and maintained under specific pathogen-free conditions at the animal facility of the Sede de Investigación Universitaria—Universidad de Antioquia (Medellín, Colombia). At the end of the experiments, euthanasia was performed using ketamine/xylazine 100/10 mg/kg intraperitoneally.

### Mice PM_10_ model and experimental design

To establish a mouse model for PM-induced airway inflammation, mice were exposed to 100 µg PM_10_ (in 50 µl PBS) per day by intranasal instillation for six days. Meanwhile, control mice were treated with the same volume of PBS.

Lungs were perfused with 1 mL PBS sterile to obtain bronchoalveolar lavage fluid (BALF). The BALF cells were evaluated by Wright's stain, and neutrophil counts were performed. The mice's lung tissues were fixed with 4% buffered paraformaldehyde (Sigma-Aldrich, USA) for 48 h and processed using standard histological techniques. After embedding in paraffin, Sections (5-μm thick) were prepared and stained with HE (Sigma-Aldrich, USA) to evaluate neutrophils infiltration for histopathological analysis. A pathologist analyzed the histopathological sections. RT-PCR was performed as described above.

### Statistical analysis

For data analysis, GraphPad^®^ Prism 8.0.1 software. (San Diego, CA, USA) the software was used. Normality was determined using the Shapiro–Wilk test. Data were analyzed using the nonparametric Kruskal–Wallis test, followed by Dunn's multiple comparisons test. Values of p < 0.05 (*) were considered significant, and values of p < 0.01 (**) and p < 0.001 (***) were considered highly significant. Only statistically significant differences are stated with asterisks in each figure. All data are expressed as the mean ± standard error of the mean (SEM).

## Results

### PM_10_ did not reduce cell viability but increased plasma membrane damage and necrosis in PMNs

**Figure 1:**
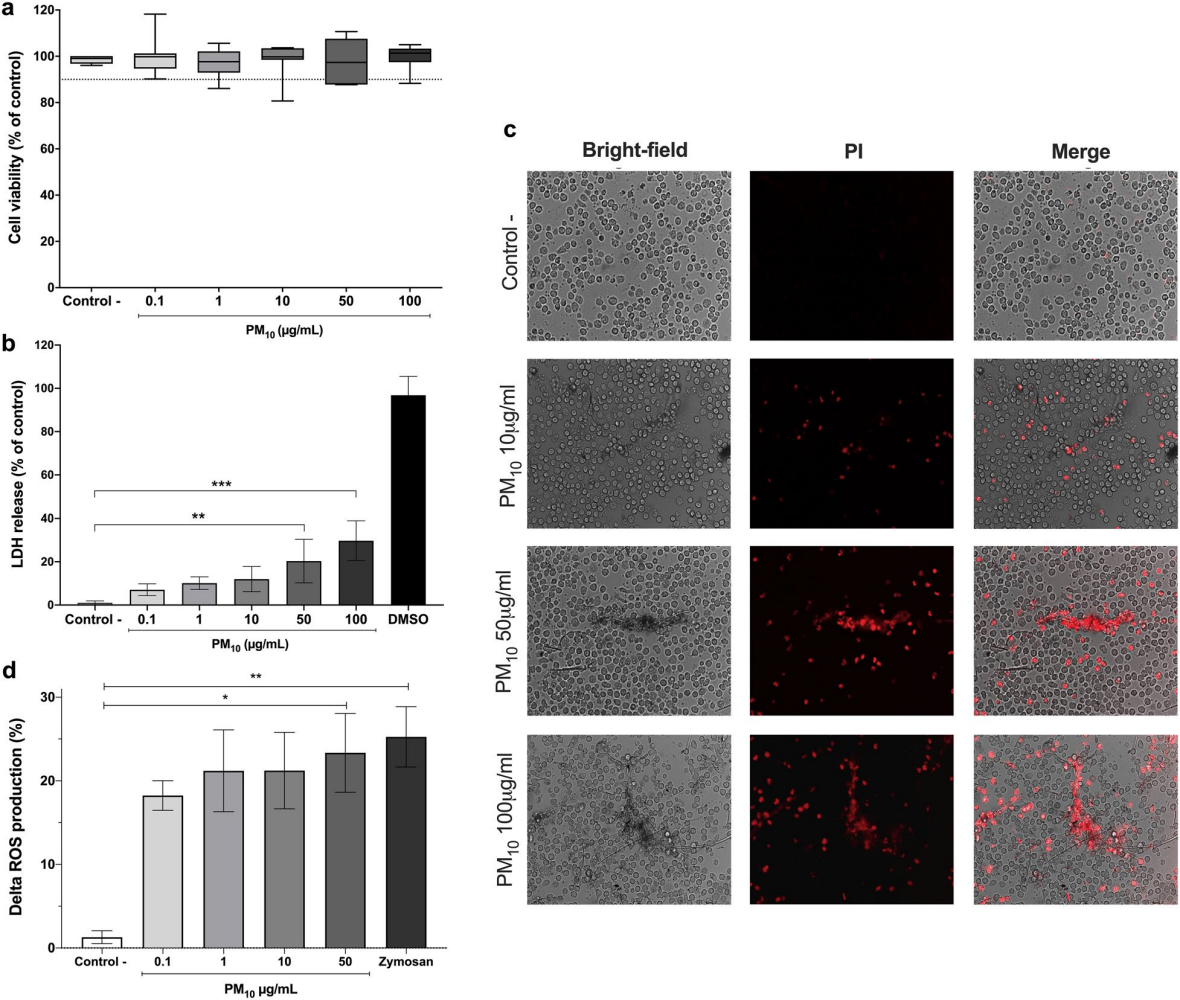
Cytotoxic effects of PM_10_ and ROS production in PMNs. (**a**) Cell viability assay by MTT and (**b**) LDH release in PMNs exposed to different concentrations of PM_10_ (0.1, 1, 10, 10, 50 and 100 µg/mL) for 5 h. Negative control (culture medium); positive control (DMSO). (**c**) Necrotic effects in PMNs exposed to different concentrations of PM_10_ (10, 50, and 100 µg/mL) for 30 min by PI staining. Photographs of each slide were observed at 200X magnification. (**d**) Intracellular ROS production in PMNs exposed to different concentrations of PM_10_ (0.1, 1, 10, and 50 µg/mL) for 30 min, determined by flow cytometry. Negative control (culture medium); positive control (zymosan). ROS levels are presented as the mean fluorescent intensity of DHR of treated cells relative to control. All data are represented as mean ± SEM of 8–14 healthy donors; *p < 0.05; **p < 0.01; ***p < 0.001, by Kruskal–Wallis analysis with Dunn's post hoc test.

Cell viability was measured by MTT assay. PMNs exposed to 0.1, 1, 10, 50 and 100 µg/mL PM_10_ for 5 h did not show a significant concentration-dependent decrease in cell viability (Fig. 1a). However, 5 h after exposure of PMNs to the different PM_10_ concentrations, LDH release was increased in the two highest concentrations (50 µg/mL: 20.33 ± 2.77% and 100 µg/mL: 29.72 ± 3.18%) compared to control cells (p < 0.05) (Fig. 1b). As shown in Fig. 1c, stimulation of neutrophils with PM_10_ showed increased red fluorescence compared to control. Thus, PM_10_-induced necrosis is confirmed.

### Exposure to PM_10_ leads to ROS formation in PMNs

ROS production in response to increasing doses of PM_10_ was determined by flow cytometry to assess their oxidative properties. An increase in ROS production was observed at all doses evaluated. However, there was a statistically significant increase from 50 µg/mL PM_10_ to the negative control (Fig. 1d).

### Exposure to PM_10_ induces IL-8 expression in PMNs

**Figure 2:**
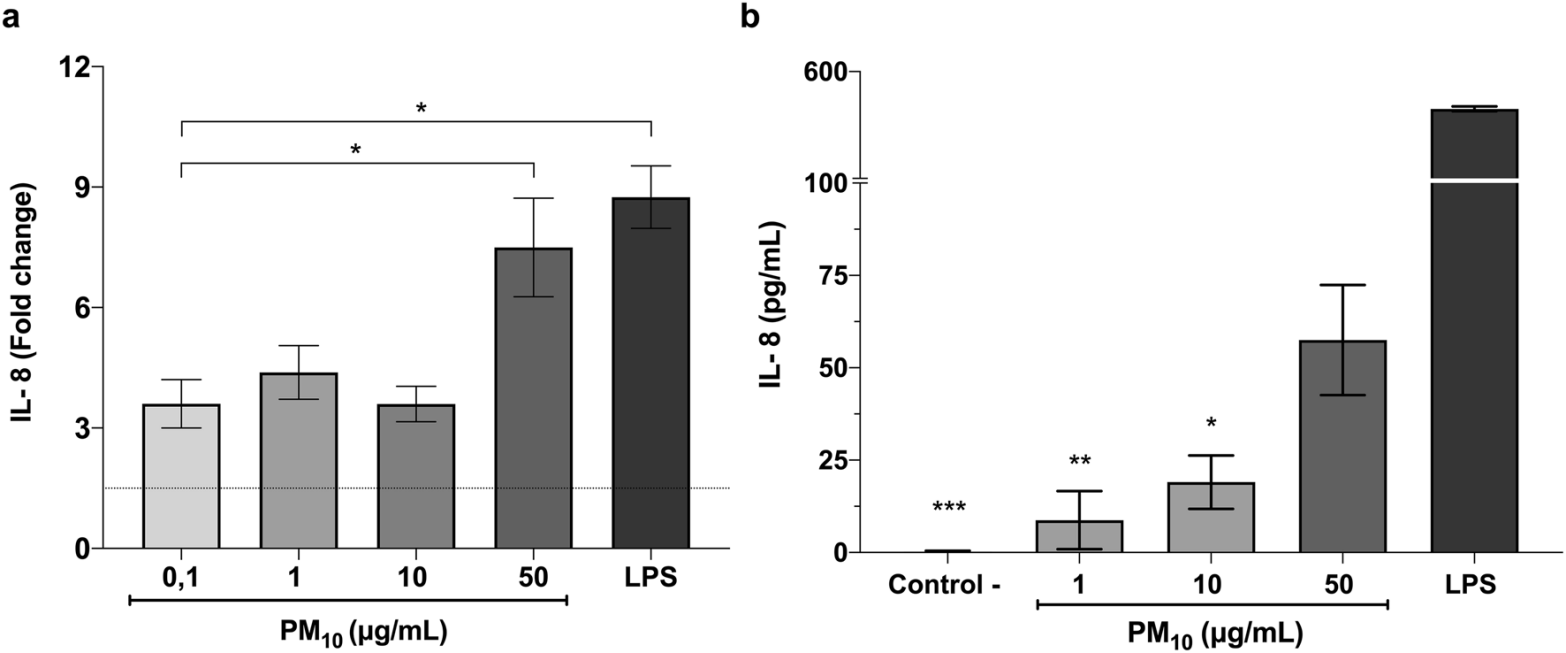
PM_10_ exposure induces IL-8 in PMNs. (**a**) IL-8 mRNA expression and (**b**) IL-8 release in PMNs exposed to different concentrations of PM_10_ (0.1, 1, 10 and 50 µg/mL) for 5 h. Negative control (culture medium); positive control (LPS). The dotted line illustrates the 1.5-fold increase over the untreated negative control. All data are represented by the mean ± SEM of 8 healthy donors. * p < 0.05; **p < 0.01; ***p < 0.001, by Kruskal–Wallis analysis with post hoc Dunn's test. In (**b**), the asterisk represents differences compared with the LPS stimulated cells.

The IL-8 expression was quantified in PMNs after 5 h of PM_10_ exposure. IL-8 mRNA expression is significantly increased (7.49-fold) in PMNs after exposure to PM_10_ (50 μg/mL) (p < 0.05; vs. control group, Fig. 2a). Regarding IL-8 protein levels in PMNs supernatants, only a slight increase was observed at 50 μg/mL compared with the untreated control (Fig. 2b).

### Exposure to PM_10_ induces the formation of NETs in PMNs

**Figure 3:**
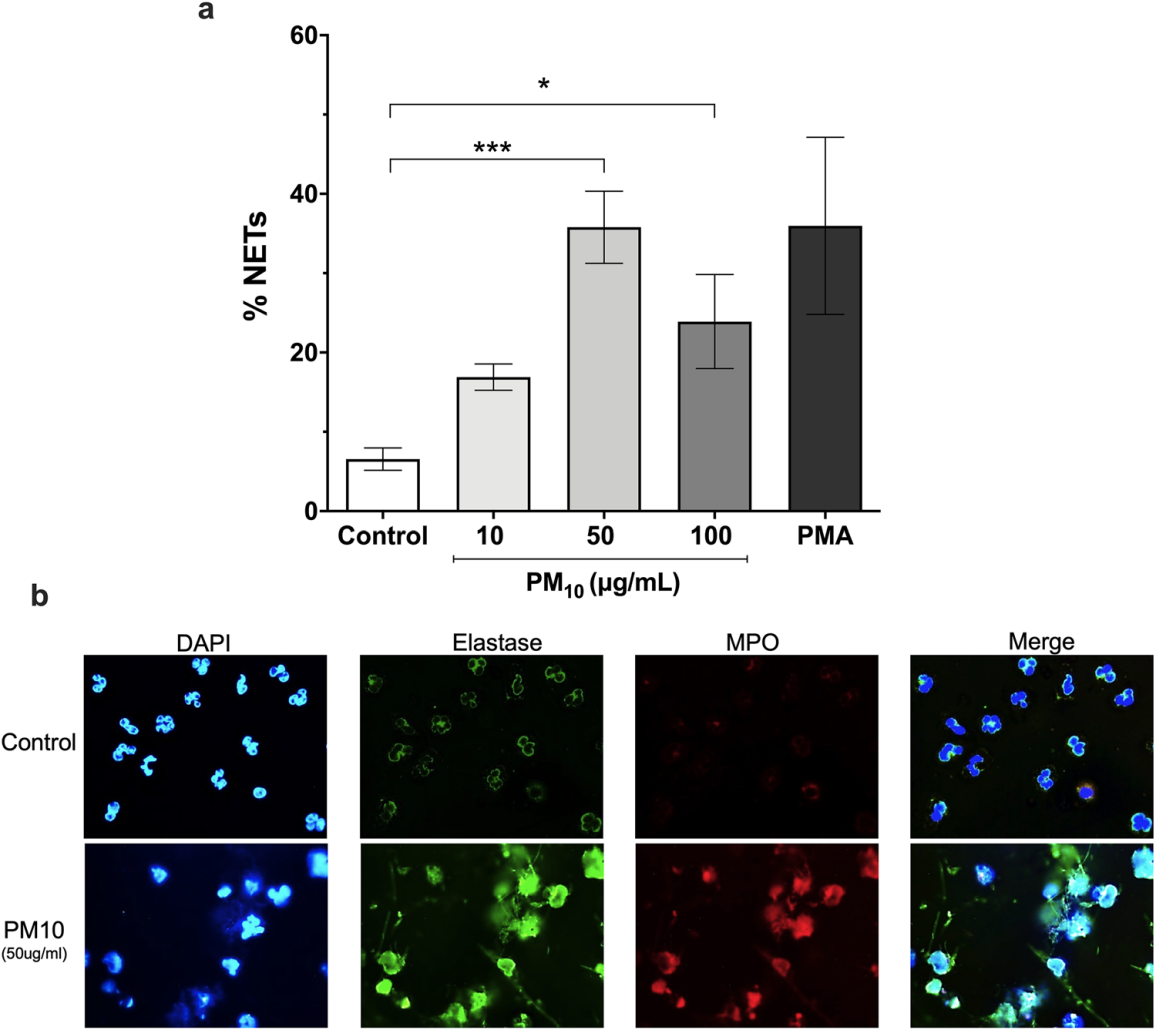
NETs formation in PMNs exposed to PM_10._ PMNs were exposed to 10, 50, and 100 μg/mL PM_10_ for 3 h. Negative control (culture medium); positive control (PMA). (**a**) NETs release was analyzed microscopically, and MPO, NE, and DNA were quantified with ImageJ software. (**b**) Representative fluorescence microscopy images of NETs. Images show colocalization of MPO (red), NE (green) with DNA fibers released (blue) from released NETs. All data are represented as mean ± SEM of 9 healthy donors. *p < 0.05; ***p < 0.001 by Kruskal–Wallis analysis with Dunn's post hoc test.

The formation of NETs in vitro in PMNs in response to PM_10_ was observed with 50 and 100 μg/mL of PM_10_ (35.76 and 23.90%, respectively) after 3 h, compared to unstimulated PMNs (p < 0.05; negative control) (Fig. 3a). Representative fluorescence photographs of NETs induction in PMNs, mediated by PM_10_, negative (unstimulated cells) and positive (PMA) controls are shown in Fig. 3b.

### Neutrophil exposure to PM_10_ causes altered expression of NETs-associated genes

**Figure 4:**
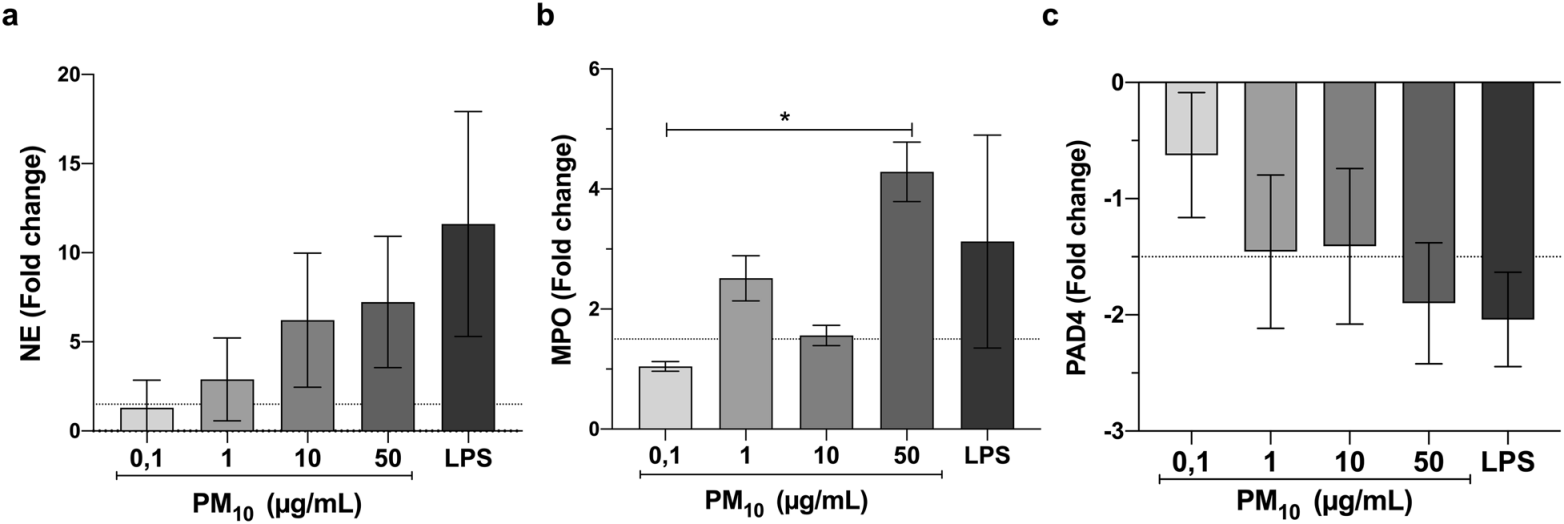
PM_10_ exposure alters NETS-associated gene expression in PMNs. mRNA expression of (**a**) NE (**b**) MPO and (**c**) PAD_4_ in PMNs exposed to different concentrations of PM_10_ (0.1, 1, 10 and 50 µg/mL) for 5 h. Negative control (culture medium); positive control (50 ng/ml LPS). The dotted line illustrates the value of 1.5-fold change compared with the untreated negative control. All data are represented as mean ± SEM of 15–18 healthy donors. *p < 0.05 by Kruskal–Wallis analysis with Dunn's post hoc test.

Gene expression of NE, MPO, and PAD_4_ was quantified in PMNs after PM_10_ exposure. MPO and NE mRNA expression increased in PMNs after PM_10_ exposure for 5 h (Fig. 4a,b). The PAD_4_ mRNA expression exhibited a negative regulation in PMNs after PM_10_ exposure (Fig. 4c).

### In vivo experiments with BALB/c Mice

**Figure 5:**
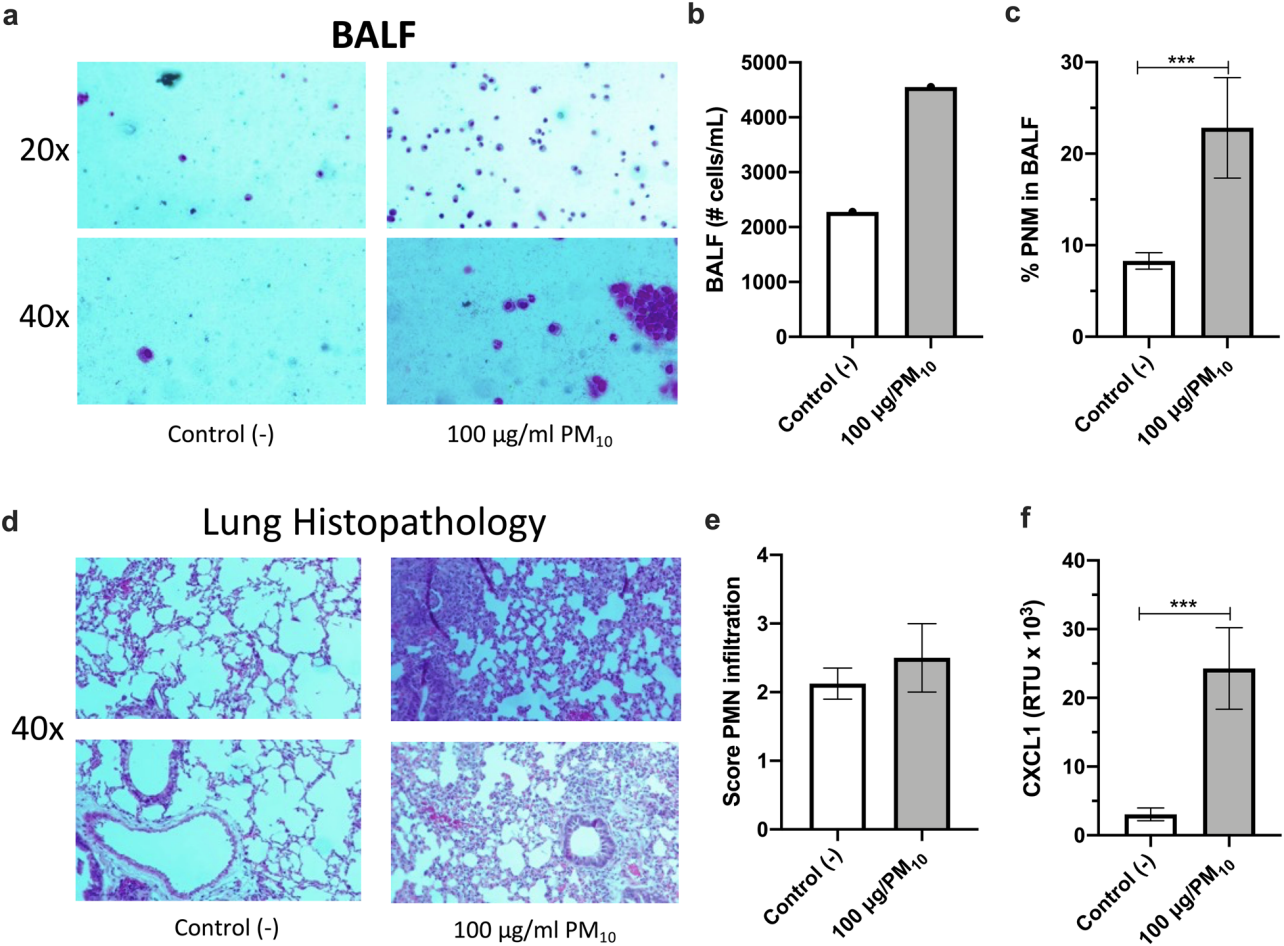
Intranasal treatment with PM_10_ increases cellularity and inflammatory infiltrate in the lung. BALB/c mice (n = 9 per group) were treated intranasally with 100 µg PM_10_ for five days. On day six post-treatment, the animals were euthanized, and bronchoalveolar lavage and lung tissue samples were collected. Wright's staining (**a**) quantification of total inflammatory cells (**b**) and quantification of PMNs in BALF (**c**) Representative images (**d**) and an inflammatory score of PMNs infiltrate (**e**) in hematoxylin–eosin stained lung sections, 1 = no infiltrate, 2 = mild infiltrate, 3 = moderate infiltrate, 4 = severe infiltrate. CXCL1 mRNA levels in lung lysate were measured by RT-PCR (**f**). All data are represented as mean ± SEM. ***p < 0.001 by Wilcoxon test.

The cellular infiltrate in the BALF of PM_10_-exposed mice was analyzed to investigate the effects on lung inflammation induced by PM_10_ (Fig. 5a). PM_10_ induced an increase in the number of total inflammatory cells (Fig. 5b) and a statistically significant increase in the percentage of PMNs (Fig. 5c) in the BALF of exposed mice relative to controls. Histopathological changes in the lungs of BALB/c mice exposed to PM_10_ were also evaluated (Fig. 5d). We found pathological alterations with inflammatory cell infiltration and moderate PMNs infiltration score in the alveoli of PM_10_-exposed mice (Fig. 5e). In addition, we observed that PM_10_ increased mouse CXCL1 mRNA expression in the lung lysate of exposed mice compared to PBS controls (Fig. 5f).

## Discussion

Inflammation caused by exposure to PM is involved in the pathogenesis of numerous diseases, such as COPD, asthma, lung cancer, cardiovascular and neurodegenerative diseases^30^. In a systematic review, we recently summarized different in vivo studies demonstrating that PM_10_ reaches the bronchi with a predominant infiltration of neutrophils^31^. Therefore, in this study, we evaluate the effect of PM_10_ on the in vitro and in vivo neutrophil-mediated inflammatory response.

In the in vitro model, no differences were observed in cell viability/metabolism when human neutrophils were exposed to increasing concentrations of PM_10_ up to 100 µg/mL (Fig. 1a). Different studies have shown that cellular exposure to PM_10_ decreased concentration- and time-dependent cell viability^32,33^. However, this is not consistent with the results of our investigation. On the other hand, two types of programmed cell death, pyroptosis, and necroptosis, are characterized by forming pores in the cell membrane and altering it, allowing the release of LDH^34,35^. In the present study, exposure to PM_10_ for 5 h increased LDH release at the highest concentrations (50 µg/mL: 20.33 ± 2.77% and 100 µg/mL: 29.72 ± 3.18%) in neutrophils (Fig. 1b). These results suggest that PM_10_ can alter the integrity of the neutrophil cell membrane, which can subsequently lead to cell death. Importantly, the assays used to analyze cytotoxicity in PMNs comprise respiratory chain activity (MTT) and membrane integrity (LDH). The latter is a much more sensitive assay, as it is based on the detection of LDH as an early event given by the loss of cell membrane integrity or damage. However, as mitochondrial metabolism is not yet affected, cytotoxicity is not detected by the MTT assay^36^.

On the other hand, necrosis, a passive and disordered cell death generated in response to physical damage or severe toxic stress, causes detrimental consequences such as induction of inflammation^37^. In our study, exposure of PMNs to PM_10_ induced necrosis, as demonstrated by increased red fluorescence of PI compared to control (Fig. 1c). Previous studies have described the necrotic effects of PM_10_^38^. In this study, PM_10_ exposure in the A549 cell line induced necrotic effects, probably due to the high metals content of PM. Therefore our data suggest that PM_10_ can generate different effects on PMNs, ultimately producing indirect toxicity.

Several factors are likely to contribute to the indirect toxic effects of PM, among which the most important may be the size and chemical composition^39^. However, there are no other studies on the cytotoxic effects of PM_10_ in neutrophils; some investigations have been carried out in vascular endothelial and epithelial cell lines of the respiratory tract, which corroborate the cytotoxic effects of PM_10_^40^. Likewise, in the BALF of murine models exposed to PM_10_, an increase in LDH concentrations has been demonstrated, evidencing epithelial injury^41^. Thus, exposure to these particles has been correlated with effects on human health^31,42^. Another study found that after a steel mill's closure, PM_10_ levels were decreased, and lower cases of respiratory admissions to hospitals were reported^42^. Interestingly, PM_10_ collected during this period produced less cytotoxicity and inflammatory response in human respiratory epithelial cells^43^. In addition, minor lung damage and neutrophilic inflammation in Sprague–Dawley rats were observed compared to PM_10_ collected while the steel mill was in operation^44^.

An additional factor that may contribute to the indirect toxic effects of PM_10_ is its ability to cause the generation of ROS, which in turn may initiate or exacerbate an inflammatory response. ROS are unstable molecules formed that play a significant role in the degradation of PM_10_ within the phagolysosome^45^. ROS species include hydroxyl radical (OH^-^), hypochlorous acid (HOCl), and hydrogen peroxide (H_2_O_2_)^45,46^. Their overabundance induces oxidative stress and reduces cell viability due to mitochondrial dysfunction, DNA damage, apoptosis, and genotoxicity^47–49^. In this study, we found that neutrophils exposed to PM_10_ presented an increase in ROS production (Fig. 1d). In this sense, our results agree with the study of Hitzfeld et al*.*^50^, who demonstrated that PM_10_ extracts collected in two German cities stimulated the production of ROS in human neutrophils. A recent report suggested that PM_10_ caused significant toxicity in the cardiovascular development of zebrafish larvae, as it led to an increase in the level of ROS and the expression of genes involved in endoplasmic reticulum stress and Nrf2 signaling pathway factors, which are essential in the regulation of cellular oxidative stress^51^.

In addition, to the indirect toxic effects of PM_10_ mediated by ROS, a recent study has shown that oxidative stress generated by PM exposure induces inflammatory responses by activating redox-sensitive transcription factors, which can lead to IL-8 expression in human bronchial epithelial cells^52^. Thus, scientific evidence has shown that IL-8 is a critical chemokine in airway inflammation induced by air pollutants^53,54^. In this study, we found up-regulation of IL-8 mRNA and IL-8 secretion in neutrophils exposed to PM_10_ (Fig. 2a,b). The secreted IL-8 mediates neutrophil recruitment into the lung and thus further amplifies inflammation in C57BL/6 mice exposed to cigarette smoke^55^. Oxidative stress and inflammation are essential factors in different respiratory and systemic diseases^56^. Dust particles can induce increased IL-8 transcriptional activity, leading to airway inflammation and exacerbation of asthma symptoms in human adults^57^. Concerning this, different studies have found that practices such as cooking with biomass are associated with increased IL-6, IL-8, and TNF-α, neutrophil infiltration, increased oxidative stress, hypertension, and tachycardia, increasing the risk of cardiovascular diseases^58,59^.

NETosis is a process that is highly dependent on ROS production^60^. In the present study, we observed that neutrophils produce NETs in response to PM_10_ than control (Fig. 3a,b). The beneficial role of NETs in infections is clear; however, new evidence has revealed a massive presence of NETs and neutrophils in the sputum of patients with cystic fibrosis and COPD^61,62^. Furthermore, NETs have recently been linked to severe clinical manifestations of COVID-19 and dysregulated immune-thrombosis in response to SARS-CoV-2^63^, strongly suggesting the role of NETs in chronic airway diseases. Likewise our findings align with a recent work that found a significant increase in the percentage of human neutrophils forming NETs measured by flow cytometry after stimulation with diesel exhaust particles (another air pollutant)^26^. Similarly, has been found that neutrophils isolated from male BALB/c mice exposed to cigarette smoke produced more NETs in response to PMA compared to control mice^64^. To note the induction of NETs seems to be decreased in neutrophils exposed to 100 µg/mL PM_10_ compared to those exposed to 50 µg/mL (without statistical differences), this may be explained because PM_10_ particles at this concentration form aggregates and thus are less accessible to cells or as shown in Fig. 1c PM_10_ is inducing another type of cell death.

Our results suggest that exposure of human neutrophils to PM_10_ resulted in altered expression of NETs-associated genes because high expression of the NE gene mRNA, MPO, was observed (Fig. 4a,b). NETs are released by a programmed form of cell death characterized by chromatin decondensation, nuclear membrane disassembly, and cell membrane rupture^65^. NE and MPO play a central role in coordinating these processes^66^. Likewise, PAD_4_ is involved in NETosis through histone citrullination and chromatin decondensation^65,66^. However, our results show a negative regulation of the PAD_4_ gene in neutrophils stimulated with PM_10_ (Fig. 4c). In correlation to our results, scientific evidence demonstrates that various activating stimuli can mediate histone citrullination and PAD_4_ requirement in NETs formation^67^. Therefore, there is a large discrepancy in the role of PAD_4_ in NETs production, with several authors reporting that the involvement of PAD_4_ depends on the type of stimulus used^68,69^. In our investigation, PM was used to induce NETs. It is well known that PM_10_ from urban areas could contain lipopolysaccharide and activate NF-κB through the MyD88 pathway, dependent on the bound LPS^70^. Thus, NF-κB has been reported to regulate PAD_4_ in mouse and human cells differentially. For example, the human cell line HL-60 treated or not with TNF-α to activate the canonical NF-κB pathway showed a significant increase in IL-8 levels (a marker of NF-κB activation) and a significant decrease in PAD_4_ levels^71^.

Consistent with the in vitro results, our in vivo findings showed that five days after intranasal instillation with 100 µg/mL PM_10_, BALB/c mice exhibited airway inflammation and increased cellularity and PMNs in BALF (Fig. 5). In lung tissue, an increase in CXCL1 mRNA expression was evident and histological analysis showed pathological alterations and neutrophil recruitment with a moderate infiltration in the lung airspaces of PM_10_-exposed mice (Fig. 5). This inflammatory response could be partly due to the ability of PM_10_ to generate cellular cytotoxicity confirmed in vitro. Furthermore, although the composition of PM varies by region, our data are consistent with previous reports on neutrophilic inflammation. In the study by Yang et al.^72^, BALF and immunohistochemical staining of lung tissues in mice 12 h after PM_10_ exposure showed visible signs of inflammation neutrophil infiltration in the airways. Likewise, CXCL1 (human IL-8 homolog) expression has been demonstrated in the BALF of C57BL/6 mice exposed to diesel exhaust particulate matter (DEP), a significant component of particulate matter that induces neutrophil-dominant inflammatory responses. In general, acute neutrophil-dominated inflammation is a mechanism of the immune system to eliminate pathological agents, including PM. However, IL-8 secretion, infiltration, and overactivation of neutrophils at sites of inflammation provide a feedback loop leading to uncontrolled inflammation that could promote the development or progression of asthma, COPD, and other diseases^73^.

It is important to note that previous studies have suggested that PM_10_ exposure induces inflammatory cytokines production and immune cells recruitment, possibly due to their high content of transition metals^74^. On the other hand, recently published experimental studies evaluating the effects of air pollutant particles on inflammatory responses have focused mainly on PM_2.5_, which includes most of the particles derived from fossil fuel combustion^75^. While these particles pose a risk to human health, coarser particles such as PM_10_ should not go unnoticed because, as our data show, they are potentially cytotoxic and inflammatory.

In conclusion, our results suggest that neutrophils are critical players in PM_10_-induced inflammatory events. These molecules induce cytotoxicity, infiltration, and activation of neutrophils, leading to ROS production, NETs release, and MPO, NE, and IL-8 expression. Future studies should evaluate the effect of PM_10_ on other neutrophils responses, including their phagocytosis and tissue migration.

## Supplementary Information

Supplementary Information.

## Data Availability

All data generated or analyzed during this study are included in this published article [and its supplementary information files].
