# Supplementary material for: Particulate matter (PM10) induces in vitro activation of human neutrophils, and lung histopathological alterations in a mouse model
Source: Sci Rep. 2022 May 9;12:7581. doi: 10.1038/s41598-022-11553-6 (PMC9083477; doi:10.1038/s41598-022-11553-6)
Supplement: Supplementary file 1 — Supplementary Information. [file 41598_2022_11553_MOESM1_ESM.docx]

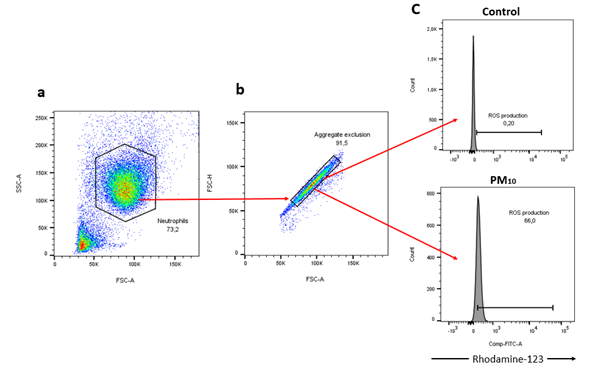


**Fig. S1** Flow cytometry gating strategy for ROS quantification in neutrophils exposed to PM_10_. (**A**) Neutrophils were separated by their higher FSC and SSC properties than other leukocyte/nucleate populations. (**B**) Aggregate exclusion was performed using a dot plot of FSC-A versus FSC-H. (**C**) Rhodamine-123 fluorescence was determined in neutrophils under control (unstimulated) and PM_10_-stimulated conditions. The increase in fluorescence resulting from ROS production is indicated by a rightward shift on the x-axis. The graphs is representative of 7 independent experiments under the same conditions.
